# Women’s Preferences and Design Recommendations for a Postpartum Depression Psychoeducation Intervention: User Involvement Study

**DOI:** 10.2196/33411

**Published:** 2022-06-23

**Authors:** Shailee Siddhpuria, Genevieve Breau, Madison E Lackie, Brynn M Lavery, Deirdre Ryan, Barbara Shulman, Andrea L Kennedy, Lori A Brotto

**Affiliations:** 1 Department of Undergraduate Medicine Faculty of Medicine University of British Columbia Vancouver, BC Canada; 2 School of Human Sciences University of Greenwich London United Kingdom; 3 Department of Obstetrics & Gynaecology Faculty of Medicine University of British Columbia Vancouver, BC Canada; 4 Reproductive Mental Health Program British Columbia Women’s Hospital and Health Centre Vancouver, BC Canada; 5 Department of Psychiatry Faculty of Medicine University of British Columbia Vancouver, BC Canada; 6 Women’s Health Research Institute British Columbia Women’s Hospital and Health Centre Vancouver, BC Canada

**Keywords:** postpartum, depression, perinatal mental health, patient engagement, women’s health, qualitative, psychoeducation, digital tools

## Abstract

**Background:**

Postpartum depression (PPD) is one of the leading causes of maternal morbidity, affecting up to 18% of Canadian new mothers. Yet, PPD often remains untreated due to numerous barriers in access to care, including location and cost. Development of eHealth interventions in collaboration with patient partners offers an exciting opportunity to fill this care gap and provide effective and affordable care to new parents across British Columbia.

**Objective:**

Our aim was to determine the content and design preferences of women previously diagnosed with PPD to inform changes to the development of a web-enabled intervention for education and management of PPD.

**Methods:**

Webpage prototypes were created to mimic the web-enabled resource using findings from completed focus group research that assessed what women want in a web-enabled support resource for PPD. A convenience sample of women aged >18 years and previously diagnosed with PPD was recruited. Feedback was collected on the content and design of the prototypes via semistructured interviews and online surveys. Qualitative, inductive analytic, and quantitative methods were used.

**Results:**

A total of 9 women (mean age 37.2 years, SD 4.8 years) completed the interview and a majority of the survey. The following 6 themes were identified: (1) inefficacy of text-heavy layouts, (2) highlighting key information, (3) clarity/understandability of the language, (4) finding support groups, (5) validation and immediate help for feelings of isolation, and (6) helpfulness and accessibility of the resource. Each theme identified elements of content or design that were either effective or may be improved upon. Most women (8/9, 89%) favored content relating to foundational knowledge of PPD, such as symptoms and management options. The layout, language, and content were found to be generally easy to understand, clear, trustworthy, and helpful.

**Conclusions:**

Six key areas were identified by women previously diagnosed with PPD, as requiring focus in a web-enabled psychoeducation program. Consistent with past research, this study also found that support and enthusiasm for web-enabled programs support PPD management as an adjunct to other evidence-based treatments.

## Introduction

### Background

Perinatal psychiatric disorders are among the leading causes of maternal morbidity and mortality worldwide, with postpartum depression (PPD) symptoms affecting up to 18% of new mothers in Canada [1,2]. Current best practice guidelines recommend management based on the severity of symptoms [3]. In mild to moderate forms of PPD, nonpharmacological treatments are recommended first, which include psychoeducation, self-care, and psychotherapies, such as cognitive behavioral therapy, prior to pharmacological treatments such as antidepressants [3,4]. In particular, psychoeducation, which involves the provision of evidence-based information, is indicated as a first-line option for education, prevention, and treatment purposes for most people experiencing mild to moderate PPD [3,5]. Despite these effective management options, many women are left untreated, often leading to poor maternal and infant health outcomes [6].

Numerous barriers, including social (eg, stigma), instrumental (eg, financial constraint), and structural (eg, lack of accessible information) barriers, prevent mothers experiencing PPD and their partners from receiving appropriate management [7]. Additionally, the COVID-19 pandemic has been shown to elevate the risks of poor mental health symptoms among pregnant and postpartum women [8,9]. With rising mental health concerns and likely further reduced access to care, the needs of individuals experiencing PPD require more attention than ever [10].

Web-enabled interventions (eg, websites) allow for the translation of psychological and other skill-based interventions via a web-based platform. Such interventions can be instrumental in targeting many barriers as they can be more accessible, affordable, and personalized, particularly in perinatal care [11-13]. With additional challenges to access during COVID-19, easily available web-enabled interventions have become even more prevalent [14]. A number of web-enabled interventions for perinatal mental health have been introduced, though with minimal engagement of end users in their development [15-17].

There is evidence that patient partnership in research designed to develop interventions results in an improved end product given that they centralize the users’ perspectives and context, as well as their lived experiences [18]. In a recent study, patient education material that was co-created with patients demonstrated a higher usability score and overall preference in comparison to education material created by only experts [19].

As a precursor to this study, Lackie et al [20] conducted focus groups to determine the unmet digital health needs of women with PPD. Participants in the focus groups believed that a web-enabled intervention could address current gaps within PPD care, including education, validation, empowerment, and accessibility [20]. Through this, the next step in this work was identified as the need to create an accessible web-enabled intervention for all women, specifically those in remote communities where in-person resources are limited.

### Objectives

In this pilot phase, we aimed to engage end users by evaluating women’s feedback on webpage prototypes of PPD-related psychoeducation content that will eventually be housed within the web-enabled intervention. We hypothesize that this prototype will be overall well-received by our participants, with feedback on various aspects including visual design and content that we will employ to improve the final product.

## Methods

### Study Population and Eligibility

The study participants were a convenience sample of women from any community across British Columbia (BC), Canada, irrespective of city of habitation or ethnicity. The inclusion criteria were established to ensure meaningful involvement within the limitations of the study. To be eligible, the study required participants to be 18 years or older; be able to read, write, and speak conversational English; have a previous diagnosis of PPD in the last 5 years; have no current PPD symptoms; and have access to a computer/device with stable internet connection. Each participant underwent a screening phone call to ensure eligibility, including a researcher-administered Edinburgh Postnatal Depression Scale (score of <12) [21]. Individuals who were eligible, as per an eligibility screening questionnaire, were invited to participate. This was a pilot study to inform the development of a web-based resource, where we aimed to recruit a small sample size for in-depth qualitative analysis.

### Recruitment

Participants were a convenience sample of women in the community recruited primarily through the following 2 methods: (1) recruitment of those who had participated in the foundational focus group research project by Lackie et al and provided consent to be contacted for future research [20] and (2) recruitment via social media advertising. We emailed each previous participant who had consented to be contacted again regarding this new phase of the study and had them respond if they wished to learn more or participate. We only reached out to each participant once unless they responded. Social media posts were made in relevant groups that catered to our target population, such as parent groups across BC. These posts provided preliminary information about the study via a study poster and a short blurb including time commitment, and participants contacted the researchers via the email provided in the post if they were interested in participating. All recruitments were completed between July and August 2020.

### Procedures

This was a convergent mixed methods design, in which the quantitative and qualitative phases of the study were conducted separately, and the findings were combined in the interpretation stage, as described by Cresswell and Plano Clark [22]. This method allows for a data validation approach, in which the open-ended questions in the interview validated the closed-ended responses in the survey [22]. To reduce participant fatigue and obtain richer data, these open-ended questions were asked in a video interview and not as open-ended questions with written responses on a survey.

#### Content Development

PPD-related psychoeducation content was initially co-drafted by a Web Development Advisory Committee (WDAC) that included researchers, psychologists, psychiatrists, community organization representatives, and patient representatives. The content was reviewed by each member thoroughly, and curated to be as relevant as possible to the target users based on recommendations and suggestions made by 2 patient representatives in the WDAC. This group virtually met multiple times to discuss the final content and design of the prototypes. Each member was reimbursed for their contribution. This content was displayed to emulate a web-enabled platform, including added colors and graphics (Multimedia Appendix 1). The pilot material included educational information, provincial resources, and instructions on finding support in the community. The education section housed foundational knowledge of PPD, including symptoms, definitions, and descriptions of management options. The resource section guided users to locate services and relevant online material. The support section had information on how users can support themselves, including creating one’s own community group or at-home self-care. The webpage prototypes were created to include only certain elements of each of these sections in order to grasp the breadth of the final website without making the material exhaustive. For example, the support section prototype only included how to create one’s own community group. It is important to highlight that these prototypes were displayed to participants in a document format made to emulate the platform, rather than a professional developed version of the web-enabled intervention.

#### Demographic Questionnaire and Content Survey (Quantitative)

All enrolled participants completed a demographic and content survey virtually from their personal devices by following an individualized link created using Research Electronic Data Capture (REDCap) tools hosted at BC Women’s Hospital in BC, Canada [23]. The demographic questionnaire collected personal information, including age, sex, socioeconomic status, relationship status, and medical history. The content survey presented webpage prototypes from each aforementioned section of the drafted content (Multimedia Appendix 2). Participants were asked to rank a list of content topics based on the quantity of content they would like to see from “not much content” to “lots of content.” Participants were also asked for their agreement on statements relating to content clarity, novelty, relevance, and usefulness, using a Likert scale from “strongly disagree” to “strongly agree.” Three binary (yes/no) questions were asked regarding the effectiveness of the visual aspects. Overall acceptability was ranked on a Likert scale from “very dissatisfied” to “very satisfied.”

Quantitative analysis was conducted with the Statistical Package for the Social Sciences, and descriptive statistics, including mean, standard deviation, and frequency counts and percentages, were calculated [24].

#### Videoconferencing Interview (Qualitative)

Interviews were virtual and semistructured, consisting of broad open-ended questions and participant-guided discussions on the webpage prototypes, and were carried out by 2 members of the research team, with 1 as the primary facilitator. The principal investigator, who is a registered psychologist, was on-call during all interviews to mediate any high-risk situations if needed. Interviews were conducted with 1 participant at a time to ensure anonymity and comfort. Each participant was given a briefing on the anonymity of the interview and was encouraged to share thoughts openly and without fear of repercussion. All participants were asked to refrain from using any personally identifying information, such as names or geographical locations, during the interview session.

An interview guide was created by the research team. The interview was initiated with an open-ended question regarding their overall feelings about the content. Each participant was also asked to compare the content with previous expectations and infer relevance and usefulness based on their lived experiences. Interviews were concluded with an open question about any final thoughts. Participants were free to discuss any thoughts relating to the intervention at any point during the interview. All interviews were audio recorded and transcribed by a professional transcriptionist (Multimedia Appendix 3).

Qualitative inductive analysis was conducted following the recommendations by Thorne [25]. In vivo coding was conducted on initial interview transcripts, whereby categories were created based on key phrases or words used frequently by participants. These categories were then combined until themes emerged from the data. The coder was a student who trained on qualitative analysis for this project, worked within a large research team at the Women’s Health Research Institute in BC, Canada, and verified the data or resolved any uncertainties. The coder was supervised by a PhD-trained researcher who had specialized training in qualitative methodology. The first author (SS) also maintained rigorous research through writing memos regarding the coding throughout the analysis process and consulted with the PhD researcher throughout the process. The interview data were also triangulated with data from the survey phase of the study. Any differences were resolved through consensus.

### Ethics Approval

Ethics approval (#H20-00931) was obtained from the University of British Columbia and Children’s and Women’s Research Ethics Board. Written informed consent was obtained from all participants prior to participating. The confidentiality of the participants was maintained at all times.

## Results

### Demographics

A total of 9 women consented to participate in the semistructured interview and questionnaire survey (Table 1). The mean age of the participants was 37 years, with an average of 2 children per participant. A majority of the participants were White European, while a minority of the participants included those with Chinese, Indigenous, and South Asian backgrounds. All participants were BC residents, married, heterosexual, and well-educated. A majority of the participants had an average household income of CAD $100,000 or more (US $78,700 or more). One participant did not complete the entire survey; thus, the analyses pertaining to the support section of the webpage prototypes were available only for 8 participants.

**Table 1:** Sociodemographic characteristics of the participants (N=9).

Variable			Value^a^
Age (years), mean (SD)			37.2 (4.76)
**Sex assigned at birth, n (%)**			
	Female	9 (100)	
**Gender identity, n (%)**			
	Woman	9 (100)	
**Sexual orientation, n (%)**			
	Heterosexual	9 (100)	
**Ethnicity, n (%)^b^**			
	Chinese	1 (11)	
	Indigenous	1 (11)	
	South Asian (East Indian, Pakistani, Sri Lankan, etc)	1 (11)	
	White European	7 (78)	
**Education, n (%)**			
	Attended some college/university	1 (11)	
	Graduated 4-year college/university	4 (44)	
	Postgraduate degree	4 (44)	
Years spent at school or in full-time study, mean (SD)			16.7 (2.69)
**Employment, n (%)^b^**			
	Full time	5 (56)	
	Part time	2 (22)	
	On maternity leave	2 (22)	
	Self-employed	1 (22)	
**Annual household income, n (%)**			
	CAD $99,999 or less (US $78,699 or less)	3 (33)	
	CAD $100,000 or more (US $78,700 or more)	6 (56)	
**Relationship status, n (%)**			
	Married	9 (100)	
Average length of current relationship (years), mean (SD)			12.6 (6.29)
Average length of longest relationship (months), mean (SD)			12.6 (6.29)
Number of children, mean (SD)			1.8 (0.44)

^a^Percentages may not equal 100 due to rounding.

^b^These data include multiple responses from individual participants.

### Survey (Quantitative) Results

#### Overall Content Preferences

**Figure 1:**
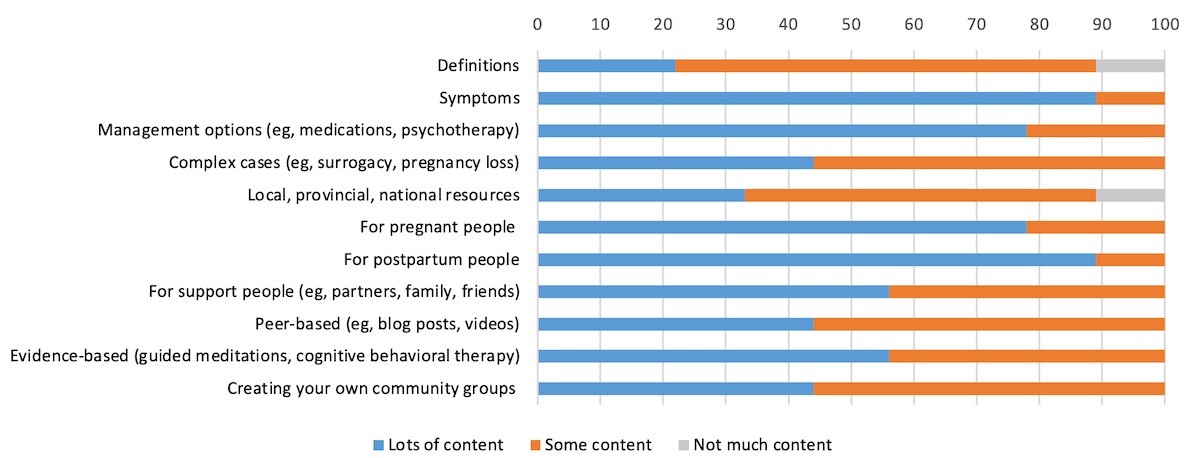
Participant preferred content ratings from “not much content” to “lots of content”.

Nearly all participants (8/9, 89%) preferred to see “lots of content” relating to symptoms of PPD and content tailored for postpartum people (Figure 1). A majority of the participants (7/9, 78%) preferred to see “lots of content” relating to management options and tailored for pregnant people. Definitions relating to PPD and information on local, provincial, and national resources were least preferred among the participants (3/9, 33%), who preferred either “not much content” or “only some content.” Other categories were more evenly distributed between “some content” and “lots of content,” such as information around peer-based supports (4/9, 44%).

#### Section-Specific Content Feedback

**Figure 2:**
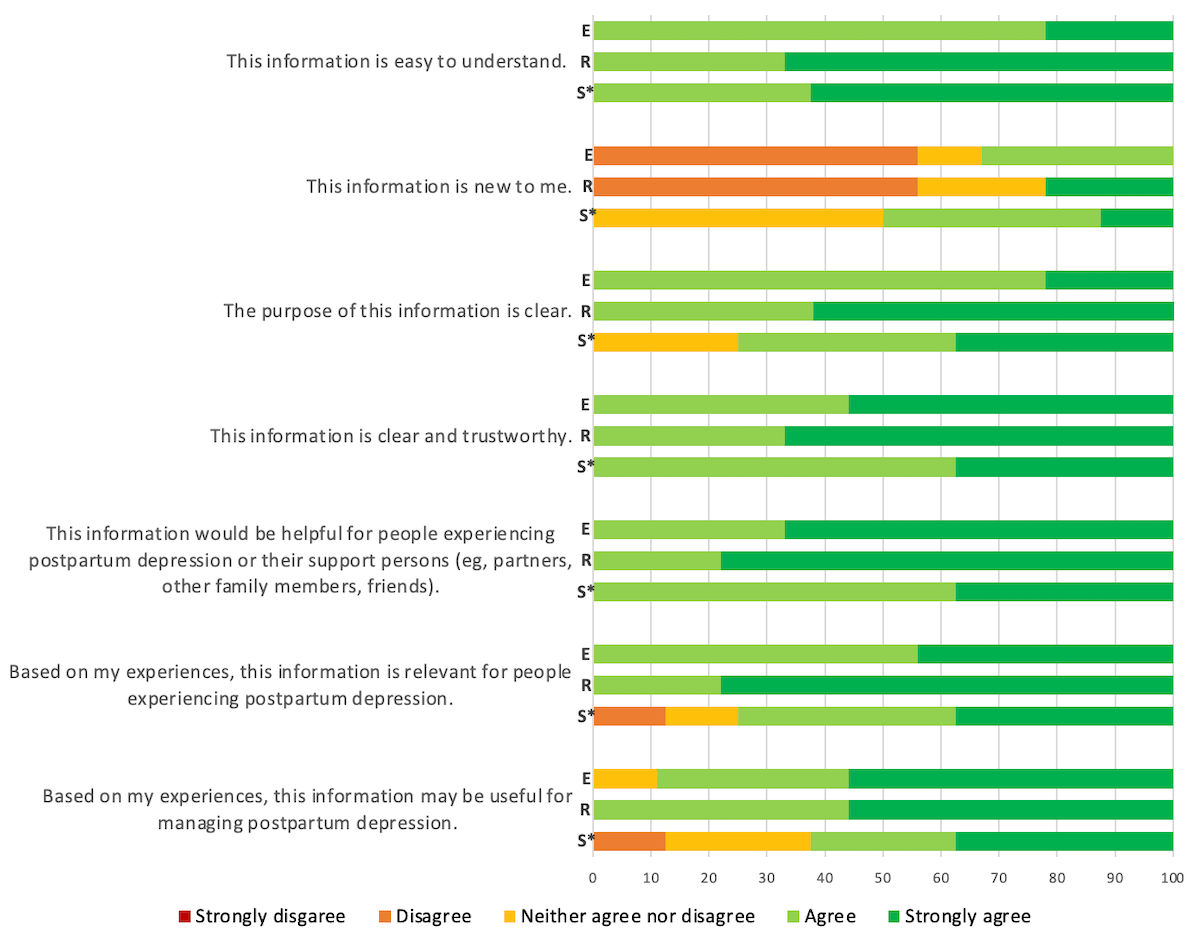
Survey responses to the excerpt content by section using a Likert scale (N=9). For the support webpage prototypes, N=8 as 1 participant did not complete this section. E: education excerpt; R: resource webpage prototypes; S: support webpage prototypes.

Nearly all participants (8-9/9, 89%-100%) either agreed or strongly agreed that the information in each webpage prototype section was easy to understand, clear and trustworthy, and helpful to people experiencing PPD and their supports (Figure 2). The information in each section was not new (ie, had pre-existing knowledge) for approximately 50% or more of the participants. All participants (9/9, 100%) agreed or strongly agreed that the purposes of the education and resource webpage prototypes were clear. A majority of the participants (6/8, 75%) agreed or strongly agreed that the purpose of the support excerpt was clear, while a few (2/8, 25%) neither agreed nor disagreed. Finally, nearly all participants (8-9/9, 89%-100%) indicated that the information in the education and resource webpage prototypes would be relevant for people experiencing PPD and helpful for its management. A majority of the participants (5-6/9, 55%-67%) found that the information in the support excerpt was relevant to people experiencing PPD and helpful for its management, while 1 participant (1/8, 13%) disagreed and a few participants (1-2/8, 13%-25%) neither agreed nor disagreed.

#### Section-Specific Language/Layout Feedback

**Figure 3:**
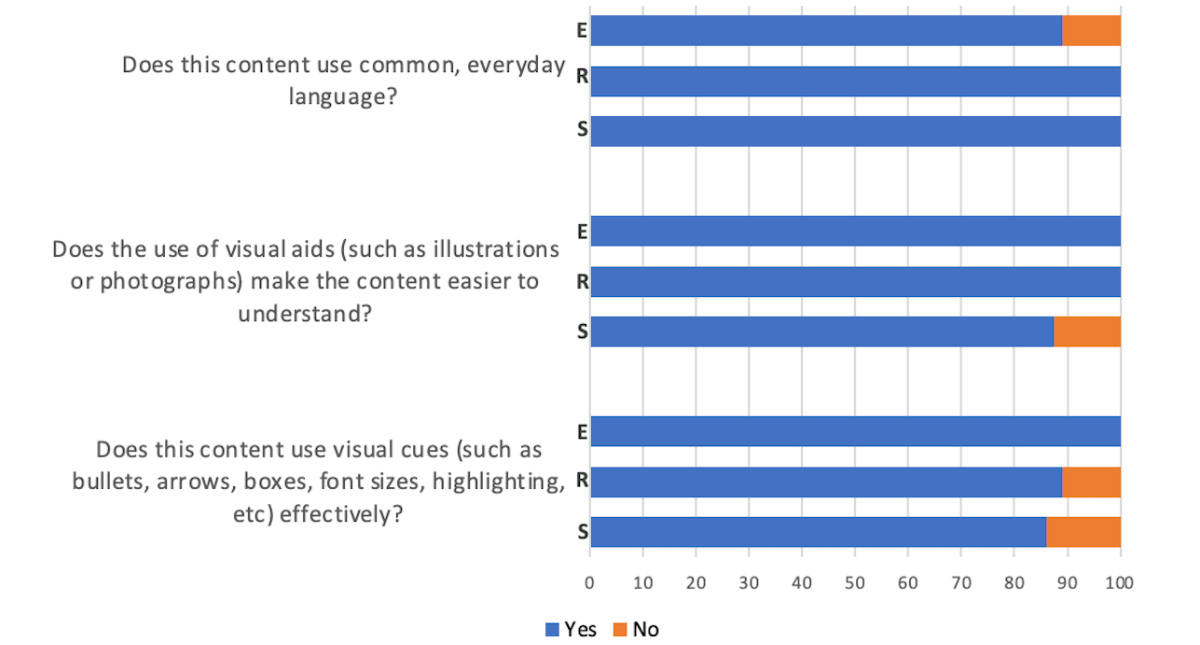
Survey responses to the excerpt language and layout style by section. E: education excerpt; R: resource webpage prototypes; S: support webpage prototypes.

All participants (9/9 or 8/8, 100%) found that common everyday language was used in the resources and support sections, while most (8/9, 89%) found that to be true for the education section (Figure 3). All participants (9/9, 100%) agreed that visual aids made the content easier to understand in the education and resources sections, while most (7/8, 88%) found that to be true for the support section. All participants (9/9, 100%) agreed that visual cues, such as font sizes, bullets, and bold style, were effectively used in the education and resources sections, while most (7/8, 88%) found that to be true for the support section.

#### Overall Satisfaction

**Figure 4:**
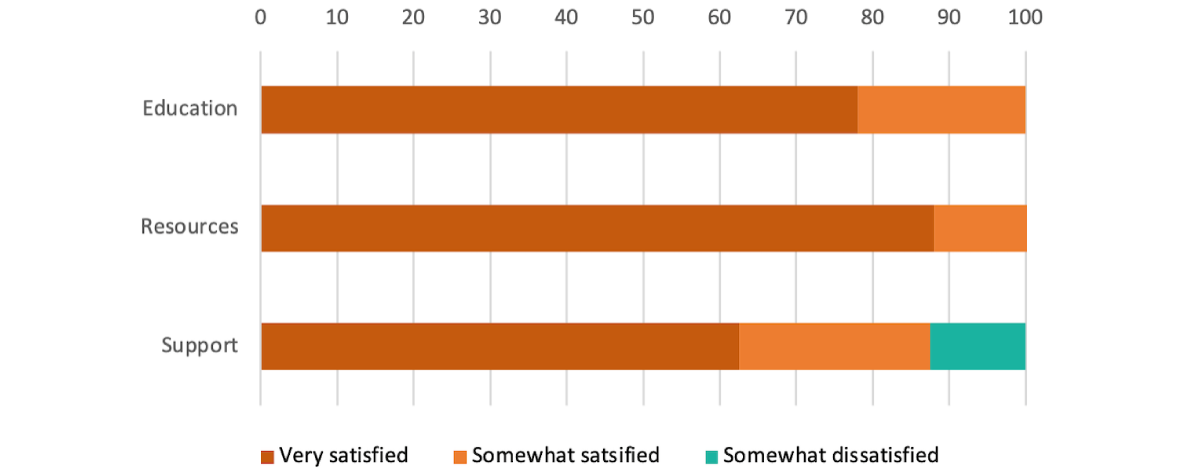
Overall satisfaction with each section excerpt.

All participants were either somewhat satisfied (1-2/9, 11%-22%) or very satisfied (7-8/9, 78%-89%) with the content and presentation of the education and resources sections (Figure 4). While most participants were either somewhat satisfied (2/8, 25%) or very satisfied (5/8, 63%) with the content and presentation of the support section, 1 participant (1/8, 13%) was somewhat dissatisfied.

### Open-Ended Interview (Qualitative) Results: Themes

Each videoconference interview ranged from 20 to 30 minutes. Using inductive analysis, we identified the following 6 themes [25]: (1) highlighting key information, (2) inefficacy of text-heavy layouts, (3) clarity/understandability of the language, (4) finding support groups, (5) validation and immediate help for feelings of isolation, and (6) helpfulness and accessibility of the resource. Both the strengths of the webpage prototypes and current gaps were discussed within each theme.

#### Highlighting Key Information

Generally, participants agreed that drawing attention to key information is pertinent for future users experiencing PPD. Participants frequently identified the need to be able to “grab something useful quickly” by using stylistic techniques such as “big bold letters,” “certain lines being highlighted,” “bullet points,” and “buttons.” Multiple respondents expressed being drawn to attention grabbing cues, and felt that this was important, especially when feeling overwhelmed.

I would increase the font…when you have depression and anxiety, it’s just very hard to focus on certain things…key words are almost better…bolding certain things maybe.

Participant aged 38 years

Really great use of bullet points, it wasn’t overwhelming, you could scan through it and kind of see what jumps out at you.

Participant aged 44 years

I like how it’s clear, and if I want to access something really quickly, there’s big bold letters and pictures.

Participant aged 37 years

Thus, while the webpage prototypes had some attention-grabbing elements, participants noted specific techniques that can be implemented throughout the resource or enhanced further on the displayed pages.

#### Inefficacy of Text and Information-Dense Layout

Most participants said that text and information-dense pages are ineffective, while some indicated that having all of the information available is also important. Given the identified need of information that draws attention, text-heavy pages contrast this need by diffusing attention to large areas of text. Multiple participants indicated that on some pages, “there’s a lot of text” and “it’s a bit too wordy,” and that it may be overwhelming, especially for people experiencing PPD.

I would want it to be very user friendly, quickly just dive into questions that you need answered, and not a lot of red tape to get through. Often when you are in the throes of postpartum, red tape is not helpful…typically don’t have the energy for it.

Participant aged 38 years

I felt like there was a lot of information there, but I actually thought it was kind of overwhelming…long paragraph, small font, it’s hard to read. I’d probably wouldn’t read it.

Participant aged 36 years

It’s a bit too wordy…from a marketing perspective, people only read like the first 10 seconds of something and then move on.

Participant aged 38 years

Generally, it was agreed that “hiding the text” and giving users the choice to view it can achieve a balance. Thus, information-dense pages can be overwhelming for users, but given the importance of the information, strategies can be used to reduce overall text.

#### Clarity and Understandability of the Language

Half of the participants found the language throughout clear and easy to follow, while the other half raised concerns that the language may be too technical at times and thus inaccessible for some individuals. Generally, participants agreed that common easy words should be used throughout the resource as it would increase the accessibility of the resource and simplify content for when amidst PPD.

I really liked how you used a common word and then also have the health or medical word for it, because for someone like me, when I go to my healthcare provider, I feel like I can understand this. You’re bridging the gap of understanding for me.

Participant aged 38 years

I like the language and visuals. They are not medical, like difficult to read. They’re just simple. And when you are in postpartum depression, you kind of need things simplified, right?

Participant aged 37 years

The language may not be easily understandable by the common population because it’s got some really big words…it’s very scientific.

Participant aged 36 years

In some ways, [the language] is quite technical…some things need to be, with medication, it needs to be in formal language but maybe others don’t need to be so formal.

Participant aged 36 years

Another participant acknowledged that English being one’s first language may limit the ability to determine the clarity of the language for users who speak English as a second or more language.

With English as my first language…I thought [the language] was quite straightforward. I found any time you had short forms or abbreviations, I could find it right away.

Participant aged 38 years

Thus, participants agreed that language should be accessible to all individuals, but complex language may be incorporated alongside it.

#### Finding Support Groups

Generally, participants stated that information on how to find existing support groups should be highlighted in comparison to how to start a support group, which may be less relevant to individuals currently experiencing PPD.

Community support group piece is probably not that relevant to me, just because I know I wouldn’t do that.

Participant aged 38 years

I feel like if somebody has PPD, it’s a lot for them to start to run a support group. For me, what would have been more helpful is being able to link with community support groups…you wanna make an equal amount or more space to finding a support group.

Participant aged 44 years

I didn’t particularly think running a support community group was at the forefront of most people going through PPD’s mind.

Participant aged 36 years

Some also noted the challenges and nuances of starting community groups.

It can feel really overwhelming to try to start your own…The idea of starting your own group feels really inaccessible.

Participant aged 44 years

To me, [the content] kind of seemed to gloss over the importance of needing qualified people to run a group like this…Having just that trained facilitator who knew how to allow people to talk in a healthy way, I think that made a huge difference.

Participant aged 38 years

Thus, participants found starting a community group lower in priority and feasibility, and suggested instead to include more information regarding finding a support group.

#### Validation and Immediate Help for Feelings of Isolation

Most participants indicated that feelings of isolation should be further addressed by sharing others’ experiences or providing immediate contacts. Overwhelmingly, participants emphasized the need for connection and validation for individuals experiencing PPD, for example, through “blog stories” or by adding a “more humane angle to [the content]” in order to remain hopeful.

Just hearing other people articulate their mental and emotional state…was so helpful…so you don’t feel so isolated. Having that basis of language of how to talk about…this is what’s happening in your brain, on a biophysical level.

Participant aged 38 years

Something that was really helpful for me was connecting with other people that had postpartum depression…who can tell you there’s hope at the other end.

Participant aged 27 years

I’d like to see some testimonies, some real-life people sharing stories…that’s really comforting to people as well…makes it more human and less clinical.

Participant aged 36 years

You want something that helps them feel like there’s some positivity at the end of this, like there’s a light at the end of the tunnel.

Participant aged 44 years

Many also noted having easy-to-find contact information for the immediate need of an individual experiencing significant distress due to PPD.

Usually you reach out for help when things are really bad…and you just need kind of an immediate thing to calm, bring that anxiety down.

Participant aged 38 years

Suicide prevention [phone] line on the top, on every page…having that resource handy and knowing someone will be there to catch you if you fall so you don’t really have to fall completely.

Participant aged 36 years

Thus, feelings of isolation were commonly experienced by participants, and it was noted that it may be helpful to incorporate relatable stories and validation messages throughout. Moreover, providing contacts for immediate need can help for when isolation feels unbearable.

#### Helpfulness and Accessibility of the Intervention

Generally, participants believed that the proposed web-enabled intervention would be a helpful and accessible resource to manage PPD initially or in early stages. Many indicated that the intervention would have been “relevant and useful” when they were managing their own PPD and would recommend it to others given the content shown.

In my own experience, I had a very difficult time accessing help, and something like this would’ve been really incredibly helpful for that. I think the fact that it’s kind of rarely available…and comes from a well-known place, like it’s here. It’s BC. It’s Canada, there’s a lot of weight of that behind it. It’s really accessible and…makes you feel not so alone in it, I think that’s the impression I was left with.

Participant aged 44 years

I wish I could just hand [the website] out to so many people I know, so when this is up and running…I love that I have something that I can refer to for people I know.

Participant aged 38 years

Useful…especially for people who have never experienced it, and don’t know how to or where to turn, or if they feel that they don’t want to reach out.

Participant aged 33 years

Thus, overall, the content shown was well received by the participants, who noted its potential usefulness not only in their past journeys, but also for others.

## Discussion

### Principal Findings

This study explored the preferences and recommendations of 9 women previously diagnosed with PPD for a web-enabled psychoeducational intervention. Based on their experiences, all participants generally believed that the presented webpage prototypes would be relevant and useful as an additional resource for education and early management of PPD. Several suggestions emerged as themes, including highlighting key information and increasing the succinctness and clarity of the language, as well as focusing the content on finding help and validation to increase the relevance, accessibility, and user friendliness of the presented prototype. This is in keeping with the findings of an earlier study, where Lackie et al determined that a psychoeducational tool could address unmet gaps in current PPD care [20]. Unlike many existing interventions, the current one is built upon patient engagement during each phase of its development [15,16,26-29].

The quality assessment of web-based tools can be divided into the following 2 major aspects: content and design [30]. Results of this study will be discussed using those 2 aspects and the factors within.

Participants expressed the most preference for the content, and in particular, information relating directly to the symptoms (ie, presentation) and management options for PPD. Several studies have identified a lack of foundational knowledge, such as recognition of symptoms, as a common and significant barrier to seeking necessary help [7,31]. In fact, directing such information to *all* individuals at risk may not just lead to early recognition but even be preventive [32-34]. In contrast, information regarding creating one’s own support group may be less relevant. The participants believed that addressing the need for immediate help, such as by helping to locate an *existing* support group, should be prioritized. In the case of PPD, when symptoms and access to care are often already prohibitive, it is important to highlight information that is likely to provide assistance in the most efficient way [31,35]. Furthermore, many participants described feeling alone during their PPD experiences and strongly suggested incorporating validating statements and relatable stories wherever possible. Even amidst social support, feelings of isolation often prevail in PPD [36]. Receiving validation and assurance, particularly from sources with an understanding of PPD, has been shown to further facilitate help-seeking behavior [37]. Validating messaging and peer stories of lived experiences are just some ways to address this need.

In regard to design, participants in this study unequivocally preferred a simple and easy-to-navigate layout with helpful features. Some specific suggestions included highlighting key information using typography techniques or having the option to view information as needed, rather than all at once. This is congruent with digital health information guidelines, which highlight the importance of enhancing accessibility through design considerations [38]. To cater to a wide range of health literacy skills (referring to an individual’s ability to locate, understand, and apply information) between users (ie, anyone with PPD or at risk for PPD), interventions must facilitate easy location and avoid high text density layouts [38,39]. As brought forth by the participants, the possible overwhelming and distressing feelings faced during PPD make it even more important to consider accessibility as the utmost priority in design across this and all perinatal mental health interventions.

### Users as Collaborators

Patient-oriented research emphasizes the importance of patient engagement to ensure high relevance, acceptance, and impact of the undertaken project [40]. This study was driven by continued involvement of relevant stakeholders, such as the WDAC (consisting of researchers, psychologists, psychiatrists, community organization representatives, and patients), to elucidate user perspective and context, ultimately informing the implementation of key ideas directly into the intervention. Particularly, each participant in this study had lived experience of PPD, thus making for a highly relevant patient group. Few of the published digital PPD resource studies have incorporated meaningful patient engagement during their development process to inform iterative changes to this degree [17,26,41]. Similar methods as described in this phase have been employed outside of PPD or in the postdevelopment phase for improvement purposes [42,43]. Yet, research has clearly identified enhanced perceived usability and consequently longer duration of use through early and persistent user involvement in development [44]. With increasing reliance of web- and mobile-enabled interventions in many domains of health, it is important to make patient engagement commonplace, as it is often both feasible and highly beneficial.

### Currently Available Resources

To date, several web- and mobile-enabled psychoeducation resources can be easily found through search engines, for perinatal mental health or PPD alone. However, few of these have been evaluated in the literature for quality assurance purposes. Of those evaluated, the quality of information still remains low, with several sources not disclosing key elements that help validate the content, such as the sponsorship or authorship [45,46]. Consistent with a previous study that utilized focus groups [20], here we found support again for well-researched and patient-centered digital resources for PPD.

### Limitations

While the findings from this study will improve the development of the web-based tool, several limitations of the study must be noted. First, a small sample was recruited due to the constraints of time and method of recruitment, which was primarily convenience sampling. Moreover, the participants were predominantly white, well-educated, and heterosexual married women. This lack of representative sampling limits the overall generalizability of the results. Additionally, participants were shown webpage prototypes in a document format created by the study team rather than a professionally developed version of the web-enabled intervention, which did not allow us to assess participant experiences with navigation. Access to the internet and internet-compatible devices was required for eligibility as we were unable to provide alternative methods of participation owing to the COVID-19 pandemic. This may have disproportionately excluded some individuals. Lastly, this study was prone to the standard limitations of online surveys and interviewing in research, including validity and reliability issues [47,48].

### Future Directions

This study adds to the existing literature calling for more evidence-based web-enabled resources for PPD. A resource, such as ours, provides information about PPD and its treatment, links users to community resources, and provides information about recovery from PPD. The next steps for our research team are to conduct a study to evaluate the effectiveness of the web-enabled resource for alleviating the symptoms of PPD in a demographically diverse group of users. Eventually, it is hoped that this will be a universally accessible resource for anyone with access to the internet and a device.

### Conclusion

This study reports many specific end-user preferences of women previously diagnosed with PPD to directly inform changes to a web-enabled psychoeducation intervention. Participants generally commended on the perceived helpfulness, reliability, and user friendliness of the webpage prototypes’ content and design. They also provided thoughtful suggestions that may enhance the impact and user experience of this resource. This study also further demonstrates the methodology and importance of involving patients at each phase of health intervention development. Overall, there is continued support and hopefulness for the potential role of this intervention as an addition to existing professional care options. 

## Appendix

Multimedia Appendix 1 Webpage prototypes as shown to participants.

Multimedia Appendix 2 Participant survey based on webpage prototypes.

Multimedia Appendix 3 Videoconference interview guide.

## Abbreviations

BC: British Columbia
PPD: postpartum depression
WDAC: Web Development Advisory Committee
