# Supplementary material for: Women’s Preferences and Design Recommendations for a Postpartum Depression Psychoeducation Intervention: User Involvement Study
Source: JMIR Form Res. 2022 Jun 23;6(6):e33411. doi: 10.2196/33411 (PMC9264129; doi:10.2196/33411)
Supplement: Multimedia Appendix 1 [file formative_v6i6e33411_app1.pdf]

Appendix 1: Webpage Prototypes

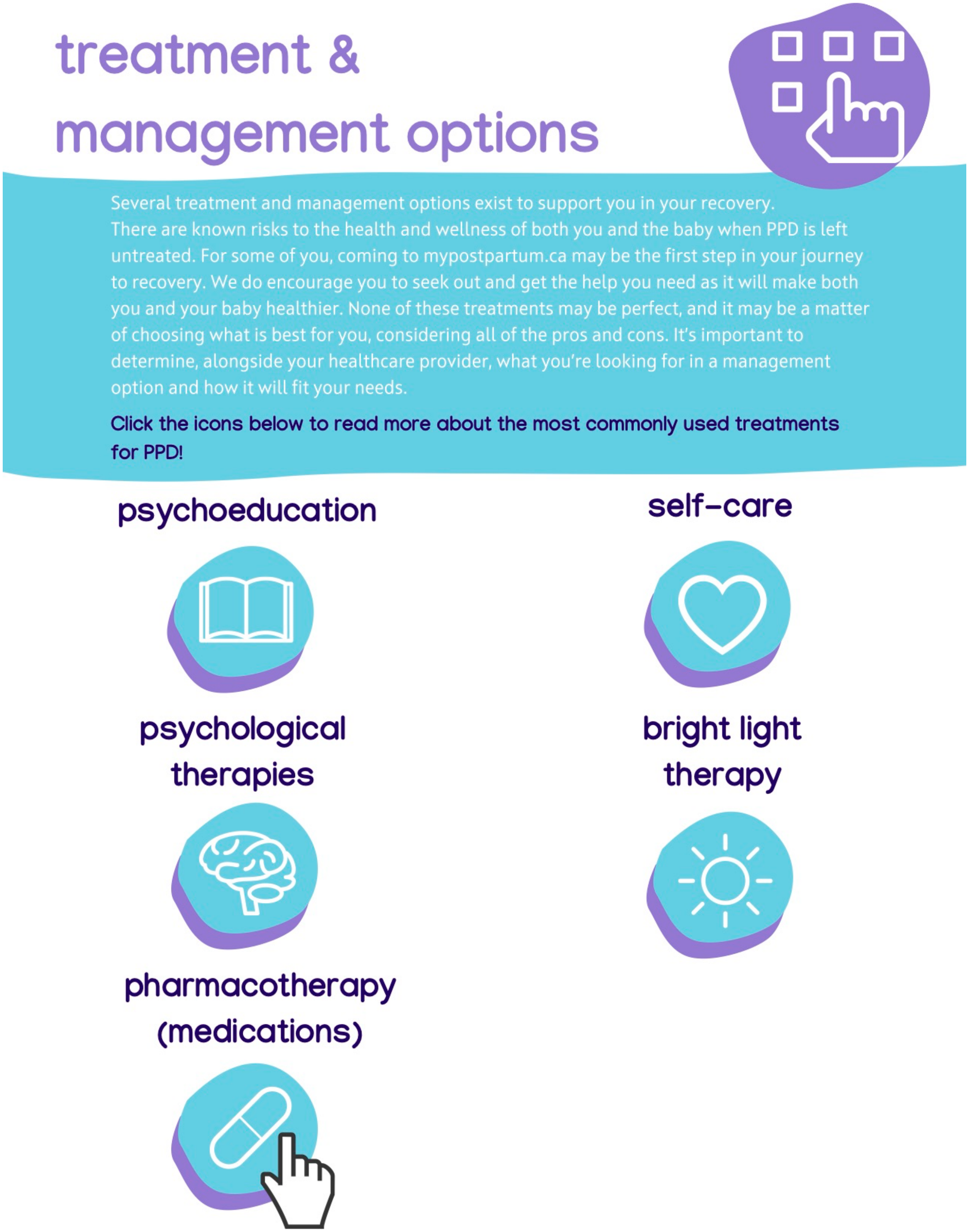

Figure 1. Education Content (page 1)

pharmacotherapy (medications)

There are several different medications that treat PPD, and they are typically prescribed based on medical guidelines and personal choice. Below are some options of medications that your healthcare provider may recommend. You should talk to your doctor or provider about which treatment is right for you and consider the medication's risks and benefits.

Antidepressants are a group of medications commonly used to treat depression symptoms, to reduce the risk of a depression recurrence (in people who have had a depression in the past), and other mental health illnesses. They work by influencing the production and function of naturally occurring chemicals in the brain that control your mood, emotions, and related behaviour. Here are some common types of antidepressants used for postpartum depression management:

Selective Serotonin Re-uptake Inhibitors (SSRIs)

SSRIs are the primary type of medications prescribed to individuals experiencing postpartum depression as they are very effective at lowering symptoms. Most SSRIs have no clear risk to breastfeeding or pregnant women. When deciding to take antidepressants, the benefits of treating the depression should be considered for you and your baby. Generally, SSRIs are taken daily, and it may take up 8 weeks to notice an effect on your symptoms.

Some examples of SSRIs include:

- sertraline (Zoloft)
- citalopram (Celexa)
- escitaloprm (Cipralex)
- fluoxetine (Prozac)

Some of these options have side effects such as weight gain or problems with sleep so be sure to talk to your healthcare provider about what will work best for you.

Serotonin and Norepinephrine Re-uptake Inhibitors (SNRIs)

Tricyclic Antidepressants

How long do I have to take medication?

Antidepressants are typically used for 6 months or longer, first to treat PPD and then to prevent a relapse (or recurrence) of mood symptoms (your healthcare provider may recommend up to a year before gradually reducing your medication for longer). Quitting suddenly can cause withdrawal symptoms or cause depression to return, so **be sure to have a discussion with your healthcare provider** to determine the best way to stop the medication, including whether it makes sense to also receive a psychological therapy.

Questions to ask your family physician about medications

1. Name & dose of your dose of medicine
2. How often you need to take it
3. How to take it - e.g., should you take it with a meal or just with a glass of water?
4. What to do if you miss a dose e.g., should you take it when you remember, or should you wait for the next dose?
5. Can I breastfeed my baby if I take this medication?

Figure 2. Education Content (page 2)

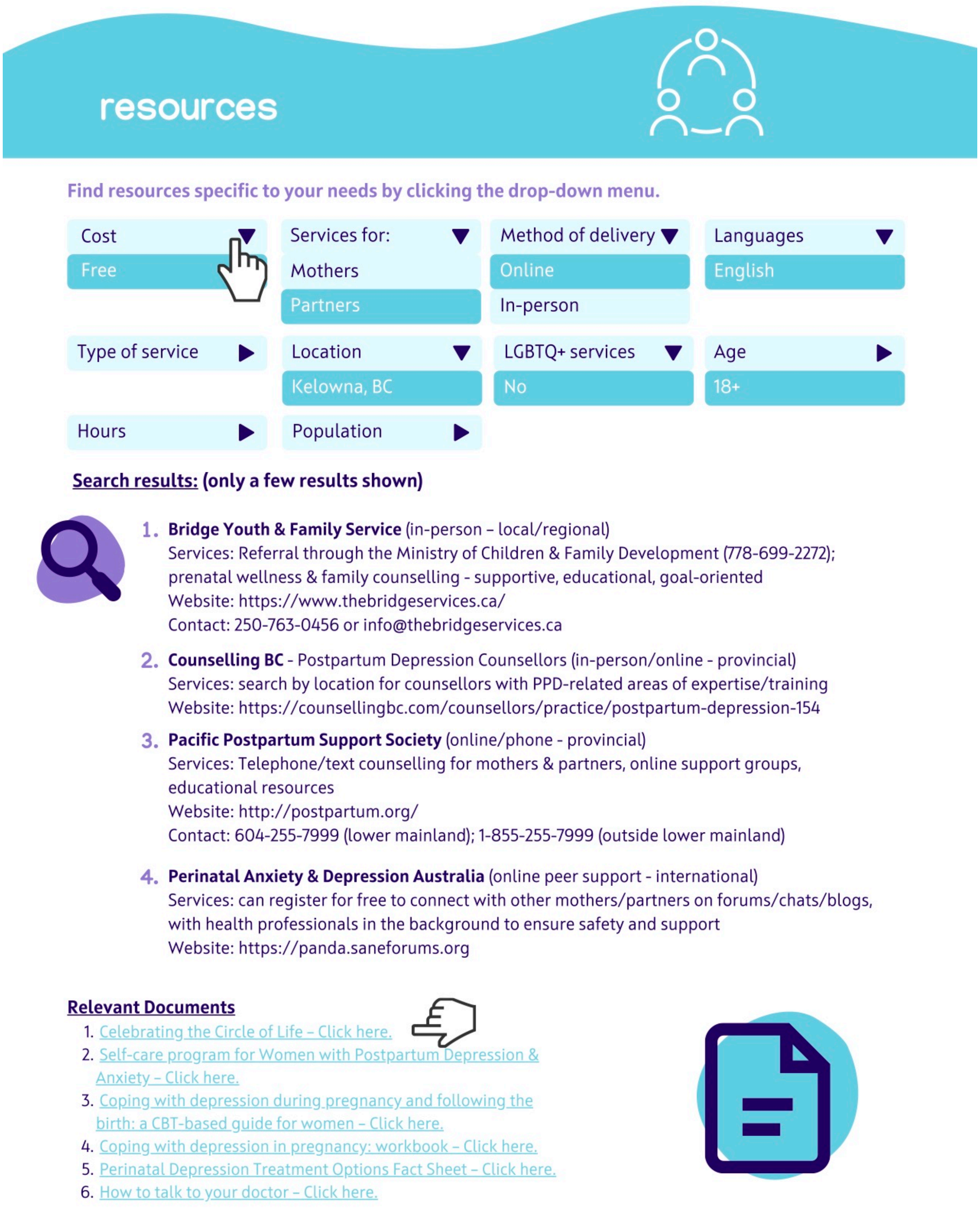

Figure 3. Resource Navigation Page

Running a Community Support Group

If you live in an urban area, you likely have resources at your fingertips to access. However, for those that live in more rural communities, resources like community support groups for perinatal mental health are often more difficult to come by. Creating your own community support group is a chance for those who have access to fewer resources, or maybe those who aren't readily able to attend their local group for whatever reason, to develop their own support network and to be a support to others.

A support group can be held either in-person or online. Benefits to hosting the groups in-person include face-to-face interaction, where it may be easier to pick up on body language and/or provide comfort. However, in-person groups can be more costly or require logistical work such as finding a low-cost/free space. Benefits to hosting the groups online include access from anywhere within the community/province/country, and low-cost (if any). As well, online groups can allow for physical distancing during the COVID-19 pandemic. However, online groups may exclude those who don't have the right technology and may limit women's willingness to share if they are talking from home.

How a community support group can be run

When you decide to run your own community support group, there are a few things that are good to keep in mind. One of those things is the comfort and safety of the other group members. To create a welcoming environment, you could (only a few examples of tips):

- Ensure there are no or minimal fees to participants

- Make sure the group is held in a child-friendly space (like a library or community centre), or check with your local community centre/school/library to see if they provide childcare services.

- Be mindful of the times you schedule the group for – if it's during the day, participants might need to arrange childcare or take time off work, but evenings might be busier and more demanding for some. If you want to determine interest before planning the group, try sending out a Doodle poll for participants to indicate their availability for certain times and dates. Create a Doodle poll here: https://doodle.com/free-poll

Figure 4. Support Content (page 1)

Advertising your group

In order to have women join your group, you will need to advertise it! Two ways you can bring participants into your group are:

Social media

Social media is a great way to advertise your group to a widespread audience. Some places you could consider advertising are: Instagram/Facebook/Twitter on your own timeline, Facebook groups, and other forums specific to perinatal mental health. You could also ask other local groups/forums to spread the word about your group in order to generate interest!

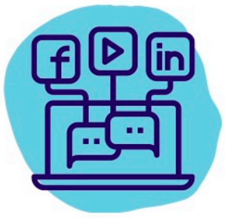

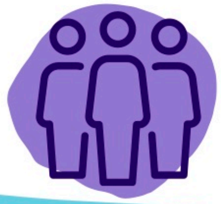

In-person

You can also advertise your groups in-person through posting flyers in community areas like libraries, community centres, coffee shops, etc. To post these flyers, you will likely need to ask permission from the establishment.

We have created flyer templates for you to advertise in both ways: click here for a social media template, click here for an in-person template.

Here is an example of one of the templates:

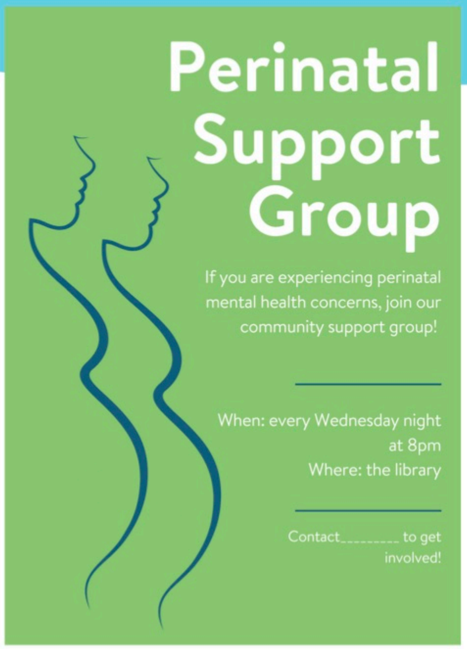

For resources on how to run community support groups:

Canadian Mental Health Association: Parent Peer Support Guide (click here)

TransCare BC of Provincial Health Services Authority: Peer Groups Start Up Guide (click here)

Free resources on designing your own support group flyer:

https://www.canva.com/

https://snappa.com/

https://www.postermywall.com/

Figure 5. Support Content (page 2)
