# Supplementary material for: Women’s Preferences and Design Recommendations for a Postpartum Depression Psychoeducation Intervention: User Involvement Study
Source: JMIR Form Res. 2022 Jun 23;6(6):e33411. doi: 10.2196/33411 (PMC9264129; doi:10.2196/33411)
Supplement: Multimedia Appendix 2 [file formative_v6i6e33411_app2.pdf]

Study Survey

Section 1: Introduction

This study aims to develop a web-based resource (i.e. website) for postpartum depression. Your feedback on the content that this resource will include will be an important step in making sure that it is useful and acceptable for people experiencing postpartum depression.

This survey will present important sections of original content created by the research team for the web-based resource. This content is based on current evidence. Section 2 will present you with an overview of the content we aim to develop. Sections 3 to 5 will present specific content, each of which will be followed by a series of questions. You will be asked to read through and evaluate this content by answering related questions.

Section 2: Overview

Before presenting you with specific content, we have created a broad overview of the content topics. Below is a flowchart of the major types of content that we plan to include on the website.

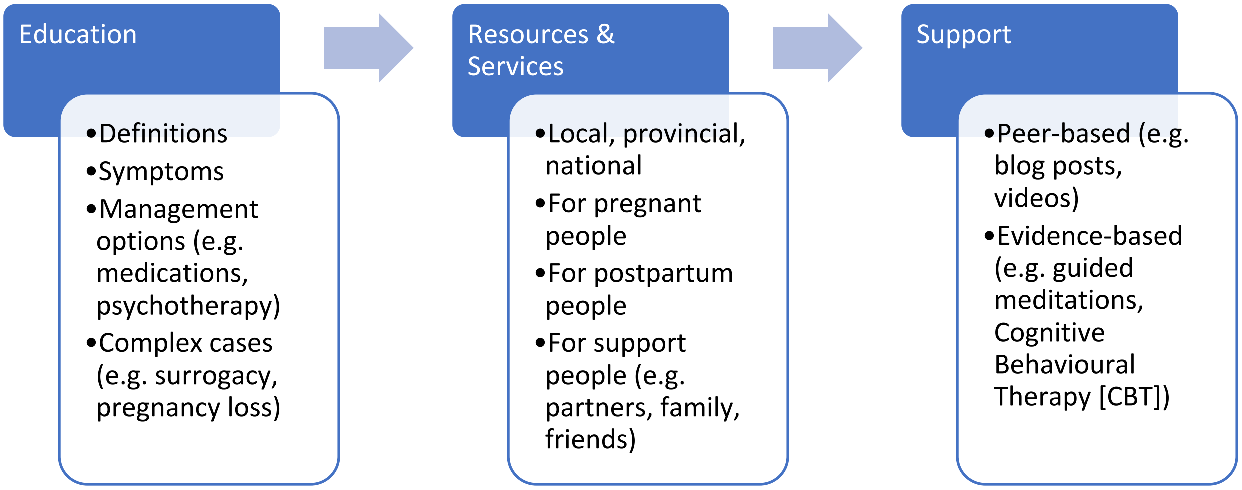

Please rate the following topics in order of how much content you would like to see from 1 to 3, where 1 is the least amount of content.
[1= not much content, 2=some content, 3= lots of content]

a. Definitions
b. Symptoms
c. Management options (e.g. medications, psychotherapy)
d. Complex cases (e.g. surrogacy, pregnancy loss)
e. Local, provincial, national
f. For pregnant people
g. For postpartum people
h. For support people (e.g. partners, family, friends)
i. Peer-based (e.g. blog posts, videos)
j. Evidence-based (e.g. guided meditations, Cognitive Behavioural Therapy [CBT])

Based on your experiences, are there any relevant categories of information/topics that may be missing?
[text box]

Section 3/4/5: Educational/Resource/Support Content

Webpage Prototypes will be shown to participants by section: Education, Resources, Support. Presentation/design would be similar to how it would be presented on the final product. (See Appendix 1)

The following questions relate to the content and visual above.

SPECIFIC: CONTENT
The following questions will be unique to each content section.

This series of questions will be based on the content presented in each section and will be generated after development of the content.

GENERAL: CONTENT
Please rate the following statements:

1. This information is easy to understand.
[1= strongly disagree; 2=disagree; 3=neither agree nor disagree; 4=agree; 5=strongly agree]
    a. If you would like to expand on your answer, please do so here:
    [text box]

2. This information is new to me.
[1= strongly disagree; 2=disagree; 3=neither agree nor disagree; 4=agree; 5=strongly agree]
    a. If you would like to expand on your answer, please do so here:
    [text box]

3. The purpose of this information is clear.
[1= strongly disagree; 2=disagree; 3=neither agree nor disagree; 4=agree; 5=strongly agree]
    a. If you would like to expand on your answer, please do so here:
    [text box]

4. This information is clear and trustworthy.
[1= strongly disagree; 2=disagree; 3=neither agree nor disagree; 4=agree; 5=strongly agree]
    a. If you would like to expand on your answer, please do so here:
    [text box]

5. This information would be helpful for people experiencing postpartum depression or their support persons (e.g. partners, other family members, friends).

[1= strongly disagree; 2=disagree; 3=neither agree nor disagree; 4=agree; 5=strongly agree]

    a. If you would like to expand on your answer, please do so here:
    [text box]

6. Based on my experiences, this information is relevant for people experiencing postpartum depression.

[1= strongly disagree; 2=disagree; 3=neither agree nor disagree; 4=agree; 5=strongly agree]

    a. If you would like to expand on your answer, please do so here:
    [text box]

7. Based on my experiences, this information may be useful for managing postpartum depression.

[1= strongly disagree; 2=disagree; 3=neither agree nor disagree; 4=agree; 5=strongly agree]

    a. If you would like to expand on your answer, please do so here:
    [text box]

<u>GENERAL: LANGUAGE/DESIGN SPECIFIC</u>

8. Does this content use common, everyday language?

[yes; no]

    a. If not, can you rewrite examples of the words/phrases from above that may need to be modified?
    [text box]

9. Does the use of visual aids (such as illustrations or photographs) make the content easier to understand?

[yes; no]

    a. If yes, can you describe any specific visual aids that stand out and why?
    [text box]
    b. If not, can you describe which visual aids are not useful and why? Would you suggest deleting those?
    [text box]
    c. Do you have any suggestions or ideas for others visual aids that can be used to improve the content?
    [text box]

10. Does this content use visual cues (such as bullets, arrows, boxes, font sizes, highlighting, etc.) effectively?

[yes; no]

    a. If yes, can you describe any specific visual cues that stand out and why?
    [text box]
    b. If not, can you describe which visual cues are not useful and why?
    [text box]
    c. Do you have any suggestions or ideas for others visual cues that can be used to improve the content?

[text box]

OVERALL ACCEPTABILITY/ THOUGHTS

11. Overall, how satisfied are you with this content and its presentation.
[1= very dissatisfied; 2=somewhat dissatisfied; 3=neither dissatisfied nor satisfied; 4=somewhat satisfied; 5=very satisfied]
    a. Why?
    [text box]

12. Do you have any other comments about this content?
[text box]

Section 5: Conclusion

1. Having read and completed sections 1-4, have you learned something that you did not know before?

[yes; maybe; no]

   a. If yes, what have you learned?

   [text box]

2. Is there anything else you would like to share?

[text box]

Thank you so much for your participation!

We will be in touch with you at your scheduled time for a brief phone interview. If you no longer wish to participate in the phone interview, send us an email at SUPPORTStudy@cw.bc.ca

.
