# Supplementary material for: Women’s Preferences and Design Recommendations for a Postpartum Depression Psychoeducation Intervention: User Involvement Study
Source: JMIR Form Res. 2022 Jun 23;6(6):e33411. doi: 10.2196/33411 (PMC9264129; doi:10.2196/33411)
Supplement: Multimedia Appendix 3 [file formative_v6i6e33411_app3.pdf]

Teleconference/videoconference Interview Script:

Introduction/Purpose

Briefly, the aim of our study is to create a web-based resource, i.e. a website, to support people with postpartum depression. You recently completed a survey where we presented you original content that our research team created for this website and asked you a series of related questions. During this interview, I wanted to ask a few more questions to explore your overall feedback and ideas about the content.

It is important to note that this conversation will be audio recorded to allow analysis of the themes that emerge and to inform changes to the website content. Your identity will remain confidential in these recordings. However, if the study investigators feel that you are a danger to yourself or others (e.g., suicidal), we will connect you with the study's Principal Investigator if you feel comfortable, or ensure that you have supports in place.

Do you consent to chatting for the next 20 minutes and having this conversation audio recorded?

[with consent]

The format for this interview will be semi-structured, meaning that I have prepared a few open-ended questions to help guide our discussion, but you are free to direct the conversation in any direction pertaining to the content or website. We have sent you an email with a copy of the content we presented to you in the survey that you can reference during this interview if you would like. If you want to take this opportunity to open that up if you haven't already. Let me know if you have not received this email and would like me to send it again.

Ground Rules

1. I understand that you may have distractions around you, but if possible, try your best to minimize background noise as much as possible. It will be easier for the audio recorder to pick up your voice if there is minimal background noise. It will also help us maintain the flow of the conversation and finish within the 20 minutes. If you need to take a break at any time to deal with kids or other pressing situations, feel free to let me know.
2. To ensure confidentiality, we will not be using your name throughout this conversation. I will ask you to refrain from using your name and/or any directly identifying information when discussing your feedback or experiences to keep this conversation confidential.
3. There are no "right" or "wrong" answers. We ask that you share your opinions honestly, and without the fear of judgement.
4. Your participation is completely voluntary, and you can choose to end the conversation at any time. You can also skip any question that you do not feel comfortable answering.

Check in #1:

1. I just want to take a moment to ask how you are doing so far? Are you feeling any distress or discomfort? If you are feeling distressed, we can certainly take a break or end the interview.

Guiding Questions

1. How did you feel about the content shown to you?
    a. Was there anything in particular you really liked or really disliked?
    b. Why did you or did you not like it?
2. Did the content we presented meet your expectations, why or why not?
    a. Is there anything you would like to change in the content that was shown to you?

Check in #2:

2. I just want to take a moment to ask how you are doing so far? Are you feeling any distress or discomfort? If you are feeling distressed, we can certainly take a break or end the interview.

3. Based on your personal experiences, what are your thoughts on the relevance of the information shown to you?
4. What are thoughts about how useful this website would be for managing depression?
5. Is there anything that you feel should be added on to the final website?

Closing

[Summary of what was discussed]

Do you have any final questions, comments, or concerns?
